# Molecular Markers Associated With Chemical Analysis: A Powerful Tool for Quality Control Assessment of Copalchi Medicinal Plant Complex

**DOI:** 10.3389/fphar.2018.00666

**Published:** 2018-06-22

**Authors:** Sol Cristians, Robert Bye, Jorge Nieto-Sotelo

**Affiliations:** ^1^Laboratorio de Etnobotánica, Instituto de Biología, Jardín Botánico, Universidad Nacional Autónoma de México, Mexico City, Mexico; ^2^Laboratorio de Fisiología Molecular, Instituto de Biología, Jardín Botánico, Universidad Nacional Autónoma de México, Mexico City, Mexico

**Keywords:** 4-phenylcoumarins, chlorogenic acid, *Exostema caribaeum*, *Hintonia latiflora*, *Hintonia standleyana*, *ITS2*, *rpl32-trnL*, *trnH-psbA*

## Abstract

The copalchi complex, *Hintonia latiflora, H. standleyana*, and *Exostema caribaeum*, is widely used in Mexico for treating diabetes and gastrointestinal disorders. The first therapeutic use for *H. latiflora* bark was registered in the “Florentine Codex” in the sixteenth century. The latest pharmacological and phytochemical studies revealed that the infusion of the leaves have hypoglycemic, antihyperglycemic and gastroprotective activities. For these reasons the monograph of the main copalchi species, *H. latiflora*, was recently added to the Mexican Herbal Pharmacopoeia. Nevertheless, quality control parameters are focused to the bark but not to the leaves. Moreover, information about other Rubiaceae species is needed. The main goal of this study was to generate molecular and chemical markers for quality control of the copalchi complex raw material. In addition, the resolution of the taxonomical ambiguity between *H. latiflora* and *H. standleyana*, as well as the testing of the molecular and chemical markers in different geographical batches, were aims of this study. The molecular markers and chemical profiles of the leaf infusions were generated considering three different populations for *H. latiflora* and separate individuals of the three species (HL, *n* = 10; HS, *n* = 3; EC, *n* = 4). The molecular markers *matK, rbcL, trnH-psbA, rpl32-trnL*, and *ITS2* were tested for their discriminating capabilities. Chemical profiles of the leaf infusions were obtained by means of HPLC analyses using chlorogenic acid and 4-phenylcoumarins as chemical markers. The concatenated sequence of the molecular markers *trnH-psbA, rpl32-trnL*, and *ITS2* clearly distinguished the three taxa, clarifying the taxonomical ambiguity of the *Hintonia* genus. Additionally, the chemical profiles allowed the unequivocal identification of each species supporting the molecular results; the geographical origin of the samples did not modify neither the chemical profiles nor the concatenated sequence of *H. latiflora*, suggesting that it is a robust identity test. The complementary use of molecular and chemical markers will assure the quality of plant material used in traditional medicine for therapeutic purposes, and should be valuable new information for the National Health authorities as a part of the Mexican Herbal Pharmacopoeia.

## Introduction

*Hintonia latiflora* (Sessé et Mociño ex DC.) Bullock, *Hintonia standleyana* Bullock and *Exostema caribaeum* (Jacq.) Roem. et Schult. are the main, and highly commercialized, species that conform the Rubiaceae section of a medicinal plant complex known as the copalchi complex (Linares and Bye, 1987). The definition of the copalchi complex is intricate because of the addition of other poorly studied Rubiaceae species such as *Coutarea hexandra* (Jacq.) K. Schum., *Exostema mexicanum* A. Gray and *Simira mexicana* (Bullock) Steyerm. and Euphorbiaceae species, mainly *Croton guatemalensis* Lotsy, *C. glabellus* L.*, C. niveus* Jacq. and *C. reflexifolius* Kunth. Also, there is a taxonomical disagreement about the identity of *H. standleyana*; some authors recognizes the existence of *H. standleyana* as a taxon (Borhidi and Diego-Pérez, 2002; Stranczinger et al., 2006); where as other specialists state that this species is a synonym of *H. latiflora* (Ochoterena-Booth, 2000; Motley et al., 2005; Martínez-Cabrera et al., 2014); morphological and molecular data were analized in both cases but chemical analysis was never used for clarify this taxonomical ambiguity.

The features that distinguish the species of this complex are the bitterness of their barks and the ancient use as antimalarial agents; nevertheless, their therapeutic efficacy cannot substitute the *Cinchona* species and must be considered as *falsas quinas* (false cinchona bark) (Anaya Dávila Garibi, 1991). In contemporary Mexico, the copalchi complex, *H. latiflora, H. standleyana*, and *E. caribaeum*, is in widespread use for treating diabetes (Mata et al., 2013), generating a great pressure over its wild populations, because all the crude drug supply is derived from wild individuals. A recent risk study of the wild medicinal species traded in the Balsas Basin, shows that *H. latiflora* is ranked in the third place of endangered species, whereas *E. caribaeum* is located in the top 50 (Beltrán-Rodríguez et al., 2017). In addition, the harvesters, motivated for the price market, increased decortication which in turn has endangered the wild populations (Martínez-Pérez et al., 2012; Reyes-García et al., 2012; Monroy-Ortiz et al., 2013).

The phytochemistry and pharmacology of *H. latiflora, H. standleyana*, and *E. caribaeum* barks are well known. Former studies have revealed both their antidiabetic properties and the nature of the active principles, which are 4-phenylcoumarins and cucurbitacins (Guerrero-Analco et al., 2005, 2007). As a strategy for the sustainable exploitation of these valuable natural resources, previous studies demonstrated that the infusion of the leaves of copalchi species exhibits noted antidiabetic action, being the major active principles chlorogenic acid and a broad diversity of 4-phenylcoumarins. This chemical mix increases the pharmacological efficacy, both hypoglycemic and antihyperglycemic, due the inhibition of α-glucosidases (Cristians et al., 2009; Mata et al., 2013). Additionally, the gastroprotective activity, which is the first therapeutic use registered in the “Florentine Codex” in the sixteenth century for *H. latiflora* bark (Sahagún, 1988), was confirmed. The efficacy of the aqueous extracts from the bark and leaves of *Hintonia* species is due to the action of 4-phenylcoumarins and chlorogenic acid that trigger endogenous sulfhydryl groups, that are important for the preservation of gastric mucosal integrity (Cristians et al., 2013).

There is no standard practice available for identifying the medicinal plant species commercialized and used in herbal products; both the consumers and the industry suffers from fraud and unethical practices, that includes substitution and adulteration of the plant material (Shanmughanandhan et al., 2016). The phytochemical and pharmacological studies of the other Rubiaceae and Euphorbiaceae species known as copalchi are not extensive, the regulation of commercialized plant materials which share the same popular names but belong to different botanical families, for example species of genus *Croton* which are related with toxicological reports (Cordell and Colvard, 2012; Sultana et al., 2018). This situation could lead to events of intoxication or absence of therapeutic efficacy, related with substitutions and adulterations. Therefore, the accurate identification of plant material is essential.

In 2013, a monograph about copalchi, *H. latiflora* bark, was added to the Mexican Herbal Pharmacopoeia in which the determining the quality control parameters for the crude drug included using TLC and HPLC as analytical methodology and 4-phenylcoumarins as marker compounds (Comisión Permanente de la Farmacopea de los Estados Unidos Mexicanos, 2013). Nevertheless, information about *H. standleyana* and *E. caribaeum*, commercialized under the same vernacular name as well as the use of leaves were not considered in the document; these aspects are important for the identification of mixtures and the sustainable resource exploitation. Recent quality control analysis shows that chemical marker compounds alone could not guarantee the identity of herbal raw material, mostly if adulterants and fillers are present; nonetheless, the concomitant use of molecular markers assures the recognition of the botanical species improving the quality of the plant material (Palhares et al., 2015; Mishra et al., 2016). This methodological procedure is not yet considered in the Mexican Herbal Pharmacopoeia; consequently, the generation of molecular markers of Mexican medicinal plants used in herbolaria is scarce.

The use of a genetic method for quality control tests should be initially supported by a library of plant material molecular markers. The loci used in barcoding analysis are the first-choice candidates for species identifications, due to its ample representation in genetic databases (e.g., GenBank) and the existence of standardized protocols for their amplification.

This study aims to provide the molecular and chemical quality control parameters for the main species that conform the Rubiaceae component of the copalchi complex, considering different populations for *H. latiflora* samples and diverse individuals for the molecular analysis of the three species: *H. latiflora, H. standleyana*, and *E. caribaeum*.

## Materials and methods

### Plant material

Leaves of *H. latiflora* were collected at three different locations in Mexico: Chihuahua State, “Entre Amigos” Ranch (579 masl), Urique Municipality in October 2014 (voucher specimens: 34439, 35140, 35141 MEXU, Mexico National Herbarium; 160156, 160153 FCME, Faculty of Sciences Herbarium); “Ex-Hacienda San Miguel” road (612 masl), Batopilas Municipality in October 2015 (160154, 160155, 160156 FCME); and in Michoacán State, “La Cocina” and “La Arena” localities (225 masl), Huetamo Municipality (voucher specimens: 131315, 131316, 131333, 131334, 131336, 131337, 131338, 131344, 131345, 131346, 131355 FCME) in July 2010. Leaves of *H. standleyana* and *E. caribaeum* were collected in Guerrero State, Tuzantlán locality, Atenango del Río Municipality in July 2010 (*H. standleyana* voucher specimens: 131342, 131350, 131351 FCME and *E. caribaeum* voucher specimens: 131339, 131340, 131341, 131343, 131347, 131348, 131349, 131355 FCME).

For molecular analysis, the individuals were labeled as follows: Urique Municipality [HL128 (34439), HL129 (35141), HL131 (160156) and HL132 (160156)], Batopilas Municipality [HL141 (160154), HL142 (160155) and HL143 (160156)], Huetamo Municipality [HL19 (131316), HL21 (131344) and HL22 (131355)], Atenango del Río Municipality [HS89 (131342), HS91 (131351), HS96 (131350), EC88 (131343), EC90 (131341), EC92 (131340) and EC93 (131339)]. For chemical analysis, the individuals were equally mixed by species and locality: HL-Urique, HL-Batopilas, HL-Huetamo, HS-Atenango and EC-Atenango.

### Molecular analysis

#### DNA extraction

DNA was extracted from leaves of the plant species using a modification of the CTAB-based method developed by Healey et al. (2014). Briefly, 10–30 mg of each sample were frozen with liquid nitrogen and pulverized using a mortar. The powder was mixed with 600 μL of extraction buffer (CTAB 2%, β-mercaptoethanol 0.03%) and incubated at 65°C for 45 min. After incubation the sample was centrifuged at low temperature (4°C) for 5.5 min at 5,000 × g and the supernatant were decanted in a new tube. The proteins, pigments and secondary metabolites were cleaned by a liquid-liquid extraction with 600 μL of chloroform:isoamyl alcohol (24:1) mixing softly for 5 min and centrifuged for 5 min at 5,000 × g (4°C). The aqueous phase was pipetted into a new tube and treated with 1 μL of RNAse A (10 mg/mL), the solution was incubated at 37°C for 15 min with periodic, gentle mixing. After incubation, 600 μL of phenol:chloroform:isoamyl alcohol (25:24:1) were added, mixing for 5 min and centrifuged for 5 min at 5,000 × g (4°C). The aqueous phase was pipetted to a new tube and 150 μL of NaCl (5M) and 900 μL of cold ethanol (95% at −20°C) were added for precipitation of the DNA. The solution was gently mixed and incubated at −20°C for 50 min. After incubation, the tube was centrifuged for 10 min at 10,000 × g (4°C); the DNA pellet was washed with 300 μL of cold ethanol (70%). The solution was swirled and centrifuged in the aforementioned conditions. Finally the DNA pellet was air-dried and suspended in 200 μL of TE buffer and stored at −20°C before use. DNA quality and concentration were quantified using a NanoDrop (Thermo Fisher Scientific, Wilmington, DE, USA) by measuring the absorbance 260 and 280 nm, and a 1% agarose gel electrophoresis. Leaves from *Coffea canephora* were used as control during all DNA extractions.

#### DNA markers amplification

DNA amplification was carried out in a first stage using the primers suggested by the Consortium for the Barcode of Life (CBOL): *matK* (F-ACCCAGTCCATCTGGAAATCTTGGTTC, R-CGTACAGTACTTTTGTGTTTACGAG) and *rbcL* (F-ATGTCACCACAAACAGAGACTAAAGC, R-GTAAAATCAAGTCCACCRCG) (Kress and Erickson, 2012) and complemented by primers used to amplify more informative chloroplast regions (Shaw et al., 2007): *rpl32-trnL* (F-CAGTTCCAAAAAAACGTACTTC, R-CTGCTTCCTAAGAGCAGCGT) and *trnH-psbA* (F-CGCGCATGGTGGATTCACAATCC, R-GTTATGCATGAACGTAATGCTC) and nuclear primer *ITS2* (F-ATGCGATACTTGGTGTGAAT, R-GACGCTTCTCCAGACTACAAT) (Chen et al., 2010; Yao et al., 2010; Palhares et al., 2015).

The PCR reactions were performed using a final volume of 30 μL for all five markers; nevertheless different reaction mixes and amplification programs were implemented. For *matK*: 0.625 U of GoTaq Flexi polymerase (Promega, Madison, WI, USA) in Colorless GoTaq Flexi buffer, 1.5 mM of MgCl_2_, 0.2 mM dNTP's (Fermentas, Vilnius, Lithuania), 0.1 μM of each primer and 10 ng/μL of DNA. For *rbcL*: 0.625 U of GoTaq Flexi polymerase in Colorless GoTaq Flexi buffer, 1 mM of MgCl_2_, 0.2 mM dNTP's, 0.1 μM of each primer and 10 ng/μL of DNA. For *rpl32-trnL*: 1 U of GoTaq Flexi polymerase in Colorless GoTaq Flexi buffer, 1.5 mM of MgCl_2_, 0.4 mM dNTP's, 0.25 μM of each primer and 10 ng/μL of DNA. For *trnH-psbA*: 1 U of GoTaq Flexi polymerase in Colorless GoTaq Flexi buffer, 0.66 mM of MgCl_2_, 0.4 mM dNTP's, 0.25 μM of each primer and 10 ng/μL of DNA. For *ITS2*: 1 U of GoTaq Flexi polymerase in Colorless GoTaq Flexi buffer, 1.5 mM of MgCl_2_, 0.4 mM dNTP's, 0.6 μM of each primer and 10 ng/μL of DNA.

The amplification was carried out in a GeneAmp PCR System 9700 Thermocycler (Applied Biosystems, Norwalk, CT, USA) using the following conditions: *matK* and *rbcL*—an initial denaturation step at 94°C for 2 min, followed by 29 cycles at 94°C for 30 s, 52°C for 40 s and 72°C for 40 s, with a final extension period at 72°C for 5 min; *rpl32-trnL* —an initial denaturation step at 95°C for 2 min, followed by 35 cycles at 94°C for 1 min, 53°C for 1 min and 72°C for 2 min, with a final extension period at 72°C for 10 min; *trnH-psbA*—an initial denaturation step at 94°C for 2 min, followed by 40 cycles at 94°C for 30 s, 55°C for 40 s and 72°C for 40 s, with a final extension period at 72°C for 5 min; *ITS2*—an initial denaturation step at 95°C for 5 min, followed by 40 cycles at 94°C for 30 s, 556°C for 30 s and 72°C for 45 s, with a final extension period at 72°C for 10 min. After amplification, the PCR products were visualized on a 1% agarose gel stained with GelRed (Biotium, CA, USA). Because some samples did not yield amplicons in the subsequent PCR reaction, the final dataset considered only a total of 46 samples. In all cases, the DNA of *H. latiflora*, specifically the individual labeled as HL19, was used as positive control.

The PCR products were sequenced in the Laboratorio de Secuenciación Genómica de la Biodiversidad y de la Salud, Instituto de Biología - UNAM, using a 3730*xL* DNA Analyzer with 96 in-capillary detection by dual-side illumination (Applied Biosystems, CA, USA).

#### Data analysis

The DNA sequences were edited using the DNA Dynamo sequence analysis software (Blue Tractor Software, North Wales, UK) and 4Peaks sequence analysis software (MRC Laboratory of Molecular Biology, Cambridge, UK). Bases with low quality or discordances between the forward and reverse strands were manually edited. The edited sequences were submitted to the GenBank (accession numbers from KX815127 to KX815139 for *ITS2*; for *trnH-psbA* from KX815141 to KX815155; and for *rpl32-trnL* from KX815158 to KX815168). The sequences of the genes *matK* and *rbcL* were discarded from the analysis because of their poor resolution and discrimination between *Hintonia* species (data not shown).

The barcoding gap analysis was performed using the Automatic Barcode Gap Discovery method (ABGD) (Puillandre et al., 2012) in order to detect a significant barcoding gap between intra- and interspecific variation and predict the finest partition of the data set into candidate species. The distance was measured by means of the distribution of pairwise differences using JC69 Jukes-Cantor model. The species identification capabilities of each molecular marker, *trnH-psbA, rpl32-trnL*, and *ITS2*, and the concatenated sequence were analyzed.

The phylogenetic analyses were performed with the concatenated sequences of the markers *trnH-psbA, rpl32-trnL*, and *ITS2* using the Maximum Likelihood (ML) statistical method by the Tamura 3-parameter nucleotide substitution model, because of its lowest Bayesian information criterion level. For the phylogeny test, we achieved a bootstrap method test with 1,000 replications. All the phylogenetic and tree assembly analyses were performed in MEGA 6 software (Tamura et al., 2013), while the trees were edited using the tree figure drawing tool FigTree v.1.4.2 (Institute of Evolutionary Biology, University of Edimburgh, UK).

### Chromatographic analysis

#### Infusion preparation

The infusions for analysis were prepared following the methodology described in previous chemical analyses of *H. latiflora* (Cristians et al., 2009). Briefly, 750 mg of milled (particle size < 2,000 μm, mesh size 2 mm) leaves from different individuals of *H. latiflora, H. standleyana* or *E. caribaeum* were pooled and extracted in 50 mL of hot water for 30 min and then filtered through Whatman No. 1 filter paper and poured into a volumetric flask and made up to 100 mL with distilled water. Before their injection on the HPLC the infusions were filtered through a 0.45 μm nylon acrodisc (Pall).

#### Chromatographic conditions for the HPLC analysis

The qualitative analysis was performed on a Waters HPLC system (Waters Co., MA, USA) equipped with a photo diode array detector (PDA), sample manager, and quaternary solvent manager. System control, data collection, and data processing were accomplished using Waters Empower 3 Chromatography Data software. For the HPLC profile of each infusion, the analytical conditions previously reported were used (Cristians et al., 2009), which consist of a Symmetry C_8_ column (Waters, series WO3251R012; 5-μm particle size, 3.9 × 150 mm i.d.); the elution system consisted of CH_3_CN-H_2_O 0.1% trifluoroacetic acid (19:81) at a flow rate of 0.4 mL/min, and the injection volume was 20 μL in all cases and each infusion was analyzed by triplicate.

#### Identification of the analytes

In order to identify the diagnostic compounds in the leaves of the Rubiaceae species, the chromatographic profiles of the infusions were compared with the retention time and UV spectra and by spiking by triplicate with standards (10 μL of standard stock solution) separated under the same analytical conditions for the following compounds: chlorogenic acid (**1**), 5-*O*-β-D-glucopyranosyl-7,3′,4′-trihydroxy-4-phenylcoumarin (**2**), 5-*O*-[β-D-xylopyranosyl-(1 → 6)-β-D-glucopyranosyl]-7-methoxy-3′,4′-dihydroxy-4-phenylcoumarin (**3**), 5-*O*-[β-D-apiofuranosyl-(1 → 6)-β-D-glucopyranosyl]-7-methoxy-3′,4′-dihydroxy-4-phenylcoumarin (**4**), 5-*O*-β-D-galactopyranosyl-7-methoxy-3′,4′-dihydroxy-4-phenylcoumarin (**5**), 5-*O*-β-D-glucopyranosyl-7-methoxy-3′,4′-dihydroxy-4-phenylcoumarin (**6**), 6′′-*O*-acetyl-5-*O-*β-D-galactopyranosyl-7,4′-dihydroxy-4-phenylcoumarin (**7**), 6′′-*O*-acetyl-5-*O-*β-D-galactopyranosyl-7,3′,4′-trihydroxy-4-phenylcoumarin (**8**), 6′′-*O*-acetyl-5-*O-*β-D-galactopyranosyl-7-methoxy-3′,4′-dihydroxy-4-phenylcoumarin (**9**), and 5-*O*-[β-D-xylopyranosyl-(1 → 6)-β-D-glucopyranosyl]-7,4′-dimethoxy-4-phenylcoumarin (**10**) (Table 1). The stock standard solutions were prepared separately by accurately weighing 10 mg, pouring into 10 mL volumetric flasks, and dissolving in CH_3_CN-H_2_O (1:3) (Mata et al., 1990, 2008; Guerrero-Analco et al., 2007; Cristians et al., 2009, 2014; Pérez-Vásquez et al., 2014).

**Table 1:** Bioactive compounds **1**–**10** identified in the leaves infusions of the copalchi medicinal plant complex.

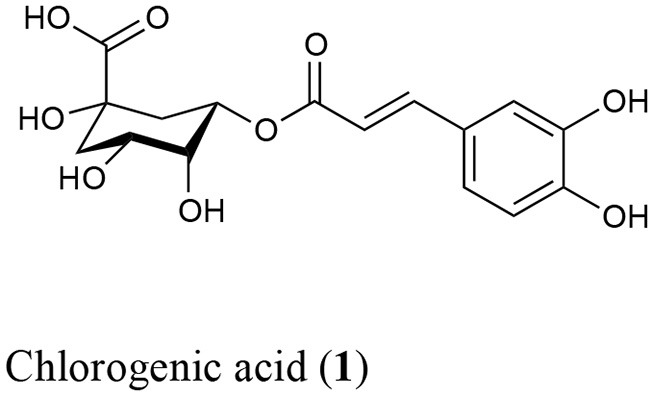		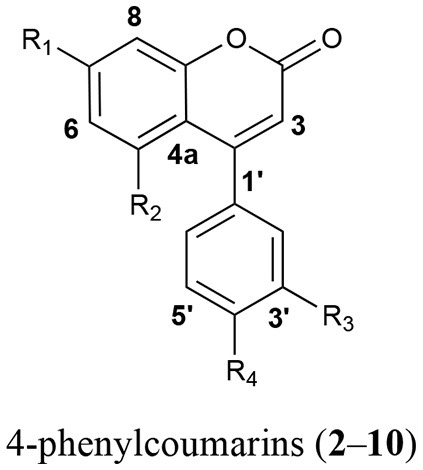		
**Compound**	***R***_1_	***R***_2_	***R***_3_	***R***_4_
2	OH	*O*-β-D-glucopyranosyl	OH	OH
3	OCH_3_	*O*-[β-D-xylopyranosyl-(1 → 6)-β-D- glucopyranosyl]	OH	OH
4	OCH_3_	*O*-[β-D-apiofuranosyl-(1 → 6)-β-D- glucopyranosyl]	OH	OH
5	OCH_3_	*O*-β-D-galactopyranosyl	OH	OH
6	OCH_3_	*O*-β-D-glucopyranosyl	OH	OH
7	OH	6″-*O*-acetyl-5-*O*-β-D-galactopyranosyl	H	OH
8	OH	6″-*O*-acetyl-5-*O*-β-D-glucopyranosyl	OH	OH
9	OCH_3_	6″-*O*-acetyl-5-*O*-β-D-galactopyranosyl	OH	OH
10	OCH_3_	*O*-[β-D-xylopyranosyl-(1 → 6)-β-D- glucopyranosyl]	OCH_3_	H

## Results

### Molecular analysis

Among the 17 plant specimens used in this study, the protocols for DNA extraction, PCR and DNA sequencing functioned for 13 (76.47%), 12 (70.58%), and 11 (64.71%) of individuals analyzed using molecular markers *trnH-psbA, ITS2*, and *rpl32-trnL*, respectively.

The nuclear marker *ITS2*, showed a maximum length of 426 bp after alignment using the algorithm Multiple Sequence Comparison by Log-Expectation (MUSCLE) (Edgar, 2004); 20 informative sites were identified plus one gap or insertion/deletion (indel) region. The chloroplast marker *rpl32-trnL*, displayed a maximum length after alignment of 785 bp, with 21 informative sites and 9 indel regions. Finally, the chloroplast marker *trnH-psbA*, had a maximum length after alignment of 302 bp, with 10 informative sites and 2 indel regions. The concatenation of the three markers generated a sequence with a maximum length of 1,513 bp, with 51 informative sites and 12 indel regions, leading to a very informative and robust concatenated sequence (Table 2).

**Table 2:**
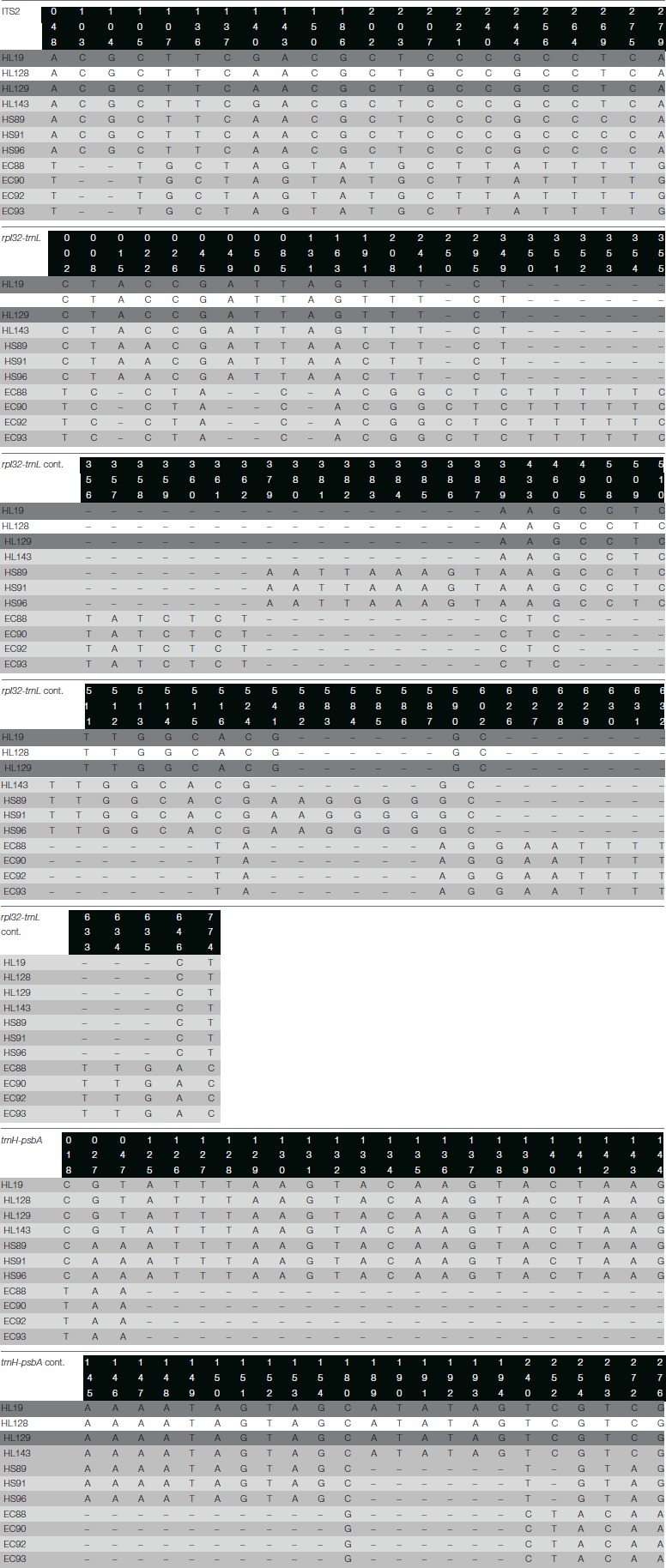
Informative sites and indel regions location of *ITS2, rpl32-trnL*, and *trnH-psbA* markers in the different species of the copalchi complex.

The barcoding gap analysis performed on the molecular markers and the concatenated sequences reveals an appropriate gap between the intra- and interspecific divergence of the *rpl32-trnL* and *ITS2* loci, whereas *trnH-psbA* displays tend to overlap both measures, lacking a clear gap; nevertheless, when the three sequences were concatenated the differences between intra- and interspecific divergence tends to restore (Figure S1). The species identification capabilities of the molecular markers and the concatenated sequence were predicted by means of the partition of the data set into candidate groups, e.g., species. The results show that *rpl32-trnL* and *ITS2* recognize up to four groups, dividing the *H. latiflora* individuals in two groups, whereas *trnH-psbA* distinguishes up to six groups generating several partitions of the three species. Finally, the concatenated sequence admits only two groups, being unable to distinguish between *H. latiflora* and *H. standleyana*.

**Figure 1:**
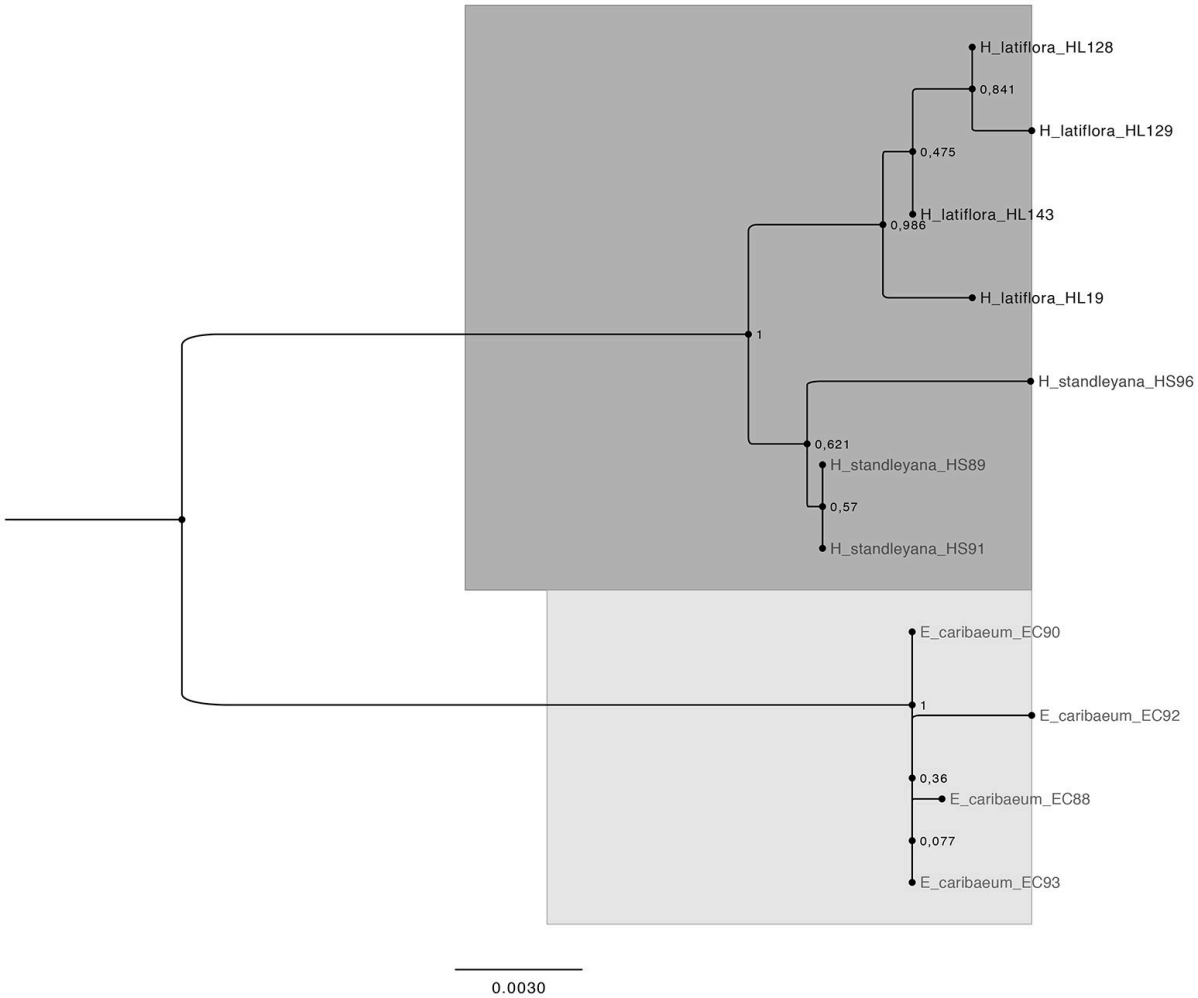
Bootstrap consensus tree generated by the Maximum Likelihood method for the concatenated markers *trnH-psbA, rpl32-trnL*, and *ITS2* sequences obtained from the three species that conform the copalchi medicinal plant complex. The dark gray highlight represents the genus *Hintonia*, the light gray highlight represent the genus *Exostema*. Evolutionary analysis conducted with MEGA 6 (Tamura et al., 2013). Numbers in the nodes are bootstrap values expressed as percentages of 1,000 replications.

The phylogenetic analysis using the concatenated sequences generates a tree in which the three species of the colpalchi medicinal plant complex were clearly separated in different clades (Figure 1). On the other hand, this analysis generated different clades for each location in which *H. latiflora* was collected, e.g., HL128 and HL129 from Urique, and HL143 from Batopilas, Chihuahua, shared the same branch that differed from HL19fromHuetamo, Michoacán, and being located in distant branch from the other *H. latiflora* individuals. Both *Hintonia* species (HL and HS) were clearly separated in two clades with a 100% of support. In addition, the genus *Exostema* (EC) is in a completely different clade, supporting the differentiation among the three taxa.

### Chromatographic analysis

**Figure 2:**
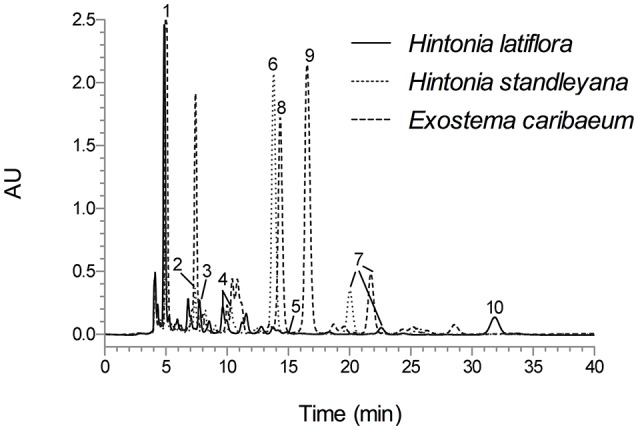
HPLC chromatogram of *H. latiflora, H. standleyana*, and *E. caribaeum* leaves infusion under optimized conditions; Symmetry C8 column (5-μm particle size, 3.9 × 150 mm i.d.) at flow rate of 0.4 mL min^−1^; mobile phase CH_3_CN-H_2_O 0.1% trifluoroacetic acid (19:81); detection wavelength 327 nm. Peak identification (t_R_, min): **1** (5.07), **2** (7.42), **3** (7.79), **4** (10.02), **5** (15.61), **6** (13.82), **7** (≈21.53), **8** (14.37), **9** (16.57), and **10** (32.19).

**Figure 3:**
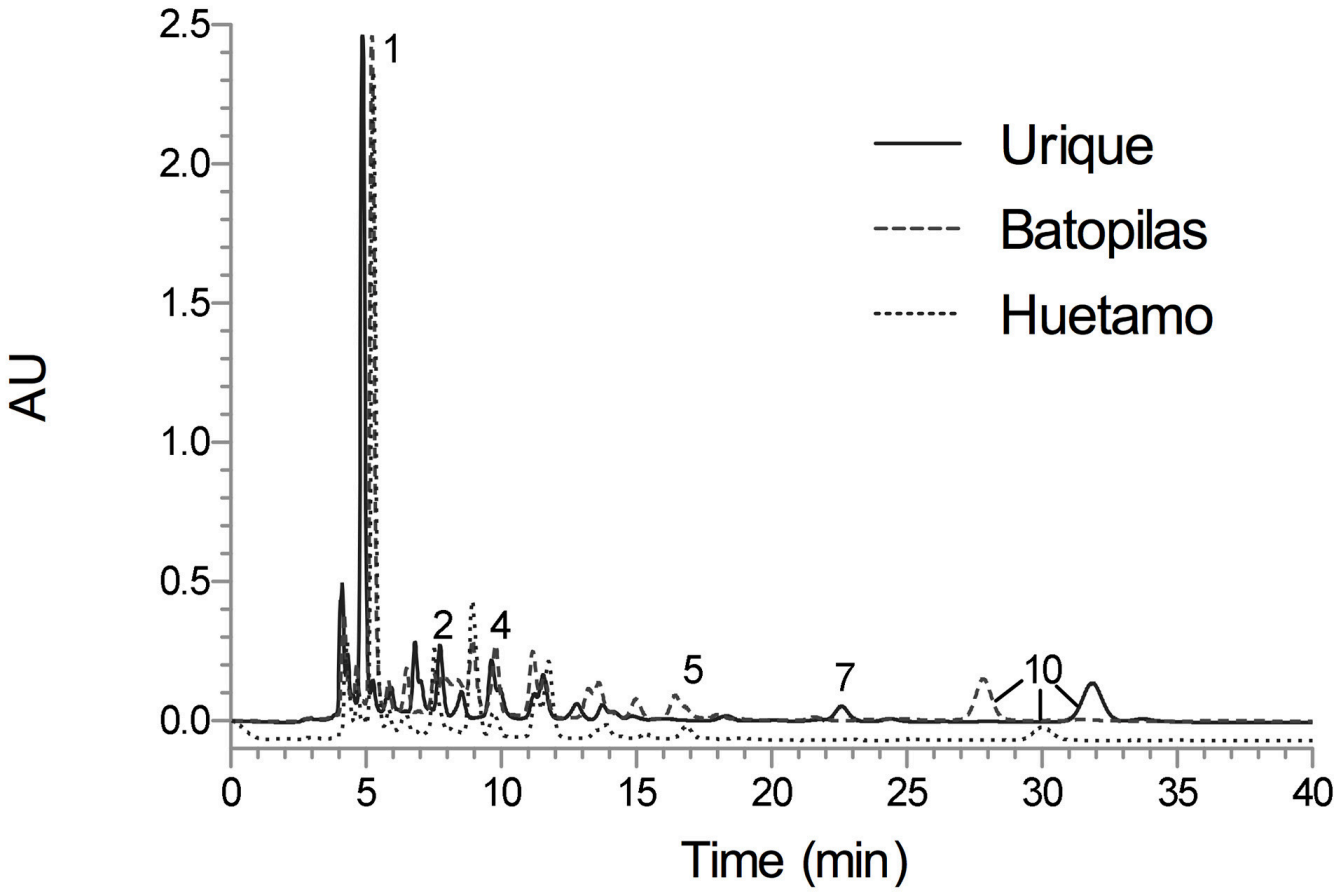
HPLC chromatogram of the *Hintonia latiflora* leaves infusions under optimized conditions; Symmetry C8 column (5-μm particle size, 3.9 × 150 mm i.d.) at flow rate of 0.4 mL min^−1^; mobile phase CH_3_CN-H_2_O 0.1% trifluoroacetic acid (19:81); detection wavelength 327 nm. Peak identification (t_R_, min): **1** (5.24), **2** (7.74), **4** (9.61), **5** (16.84), **7** (22.61), and **10** (≈31.84).

The analytical method was applied for the qualitative chemical comparison of the Rubiaceae species that conform the copalchi complex (Figure 2) and the different localities where *H. latiflora* was collected (Figure 3).

The chemical profile allowed the unequivocal identification of each medicinal species (Figure 2); in the *H. standleyana* leaves infusion, the main compound was **6**, a 4-phenylcoumarin, whereas in *H. latiflora* and *E. caribaeum* was the chlorogenic acid (**1**). Nevertheless, in the leaf infusion of *E. caribaeum* only acetylated 4-phenylcoumarins (**7**–**9**) were identified.

A previous study showed that the chemical composition has not significant differences between different populations (Cristians et al., 2014); in this research a third locality was added to this comparison, validating the same result. Chlorogenic acid (**1**) was the main compound and the mixture of 4-phenylcoumarins (**2**, **4**, **5**, **7**, and **10)** displayed the same profile in all the batches; only compound **10** had different retention times due to column overuse (Figure 3).

## Discussion

The safety and efficacy of medicinal plants relies in the quality control of plant material (Cañigueral and Vila, 2005). There are several methodological approaches for fulfilling the accurate identification of the medicinal plant species but the phytochemical analysis is the leading choice, being part of contemporary regulatory documents and pharmacopeias. The chemical variation related to the physiological influence, intraspecific differences (chemotypes) and storage conditions could jeopardize the correct identification of plant species (Techen et al., 2014; de Boer et al., 2015; Ivanova et al., 2016). Additionally, the presence of a chemical marker may be present in closely related taxa that may contain harmful metabolites and threaten the safety of the consumers (Palhares et al., 2015).

The use of molecular tools for species authentication, e.g., barcoding or molecular markers, has been used recently as a complementary approach and has been tested for the identification of adulterants, substitutes and fillers in herbal formulations providing a reliable quality control of the plant material (Sucher and Carles, 2008; Techen et al., 2014; de Boer et al., 2015; Palhares et al., 2015; Ivanova et al., 2016; Mishra et al., 2016; Raclariu et al., 2018). The recent British Pharmacopoeia includes the use of a DNA-based method for the identification of *Ocimum tenuiflorum* (Heinrich and Anagnostou, 2017).

The DNA degradation related with post-harvest or manufacturing processing of plant material is the main limitation of the molecular methodologies (Techen et al., 2014; Heinrich and Anagnostou, 2017; Raclariu et al., 2018). In addition, the extraction of pure and high molecular weight DNA for certain species and crude drugs, the affinity of the primers and the presence of secondary metabolites that inhibits the PCR must be considered during the molecular analyses (Techen et al., 2014; de Boer et al., 2015; Raclariu et al., 2018).

The plants used in Mexican traditional medicine are under-represented in the Mexican Herbal Pharmacopoeia; nonetheless, the copalchi, *H. latiflora* bark, was included since 2013 but the other Rubiaceae species that conform the medicinal plant complex and the leaves as crude drug substitute were not considered.

This omission compels us to develop a HPLC method for the chemical analysis of the copalchi complex. Harmonizing with the copalchi monograph in the Mexican Herbal Pharmacopoeia, compounds **1**, **3**, and **10** are the chemical markers for the identification of *H. latiflora*, while 4-phenylcoumarins **2** and **6** define the chemical identity of *H. standleyana*; finally, the acetylated 4-phenylcoumarins **8** and **9** are restricted to the *Exostema* species, allowing their unequivocal identification.

The use of molecular methodologies for the quality control of plant material intended for therapeutic uses are neglected in Mexico, leading to the virtually inexistence of the molecular databases of medicinal plant species. We suggest the use of the loci proposed by CBOL as molecular markers for quality control of medicinal plants. However, their use (e.g., *matK* and *rbcL*) was not always informative (Roy et al., 2010), not even concatenated, at least for the Rubiaceae species analyzed (data not shown). On the other hand, the intergenic spacer, *trnH-psbA*, is highly variable, being a successful marker for a wide range of angiosperms; *rpl32-trnL*, also an intergenic spacer, had low hybridization rates when compared with other regions of the chloroplast genome, being also variable and a good candidate for phylogenetic studies (Shaw et al., 2007). The second internal transcribed spacer of nuclear ribosomal DNA, *ITS2*, represents one of the most suitable region for DNA for quality control of medicinal plants (Chen et al., 2010; Palhares et al., 2015; Mishra et al., 2016). The use of other chloroplast markers (Shaw et al., 2007) in combination with nuclear ones (Chen et al., 2010; Yao et al., 2010) are much more advantageous.

We analyzed the DNA sequences, *trnH-psbA, rpl32-trnL*, and *ITS2*, with two methods: barcoding gap and phylogeny. The barcoding gap analysis struggles with the delimitation of the three taxa, regardless of the appropriate gap between the intra- and inter-specific divergence of the concatenated sequence, it cannot separate the *Hintonia* species. The application of these molecular markers as barcodes could be only achieved using *rpl32-trnL* and *ITS2* alone; however, they tend to show a high intraspecific divergence that could misidentify different individuals as separate species. It is important to point out that the ABGD program used can suggest the existence of different species, but it is not definitive proof and must be used along with other characters that make the species delimitation more reliable (Puillandre et al., 2012).

The phylogenetic approach generates a tree that clearly separates the three Rubiaceae species that conforms the copalchi complex: *H. latiflora, H. standleyana*, and *E. caribaeum*; the concatenated *trnH-psbA, rpl32-trnL*, and *ITS2* sequence reinforce the molecular evidence in order to recognize *H. latiflora* and *H. standleyana* as two different taxa (Borhidi and Diego-Pérez, 2002; Stranczinger et al., 2006). The approach of species identification with a Maximum Likelihood tree profile does not necessarily depend on the barcoding gap but on the coalescence of conspecific populations and the monophyly of species (Wiemers and Fiedler, 2007).

The delimitation among closely related species using DNA barcodes is not always clear. The acquisition of a significant barcoding gap improves by obtaining quality samples for analysis (e.g., number of individuals and geographical amplitude) as well as by combining those results with other data in order to create a solid taxonomic foundation (Meyer and Paulay, 2005; Wiemers and Fiedler, 2007). The signature species of the complex, *H. latiflora*, has an extended latitudinal geographical distribution in Mexico; our study indicates the differentiation between populations from the opposing northern (Chihuahua) and southern (Michoacan) limits. Nonetheless a more exhaustive collection effort must be made along the distribution gradient (Sierra Madre Occidental) in order to test the discriminatory capabilities of the proposed molecular markers.

The results obtained by chemical analysis coincided with those obtained from molecular marker analysis for the three Rubiaceae species. The unequivocal recognition of each taxon was attained by both identity tests, no matter the geographical origin of the samples. Moreover, the qualitative chemical analysis performed revealed unique chemical markers and fingerprints for each copalchi complex species, achieving the main goal of this study. Notwithstanding these results, further work is needed in order to increase the consistency of preparations from copalchi complex species. For example, to set doses for the cure of ailments using these species, quantitative analyses of the active ingredients for comparison between selected batches must be developed, as reported in previous studies (Cristians et al., 2014; Pérez-Vásquez et al., 2014). The overall results agreed with the findings of Sharma et al. (2012), one of the few reports that combine molecular analysis based on phylogeny and chemical studies using TLC for the quality control of a Mexican medicinal plant, *Galphimia glauca*.

The complementary use of chemical and molecular markers for quality control achievement of the copalchi complex and other plant material should be tested not only in commercialized crude drugs but also in the herbal preparations. The identification of adulterants, fillers and/or substitutes could be accomplished only if the molecular databases of medicinal plants are enriched with more studies. A national initiative for the establishment of a DNA herbal reference library is mandatory; this strategy is developing in countries like India, where their herbal market is vast and complex (Mishra et al., 2016).

One of the key objectives of the 2014–2023 World Health Organization's Traditional Medicine strategy is to “promote the safety, efficacy and quality of traditional medicine by expanding the knowledge base, and providing guidance on regulatory and quality assurance standards” (World Health Organization, 2013). Given this scenario, in which Mexico is actively involved because of their widespread use of herbolaria, it is crucial that these molecular and chemical complementary analyses are adopted as components of the Mexican Herbal Pharmacopoeia. Their compliance will assure the safety, efficacy and quality of plant material used in herbolaria.

## List of compounds studied

chlorogenic acid (**1**); 5-*O*-β-D-glucopyranosyl-7,3′,4′-trihydroxy-4-phenylcoumarin (**2**); 5-*O*-[β-D-xylopyranosyl-(1 → 6)-β-D-glucopyranosyl]-7-methoxy-3′,4′-dihydroxy-4-phenylcoumarin (**3**); 5-*O*-[β-D-apiofuranosyl-(1 → 6)-β-D-glucopyranosyl]-7-methoxy-3′,4′-dihydroxy-4-phenylcoumarin (**4**); 5-*O*-β-D-galactopyranosyl-7-methoxy-3′,4′-dihydroxy-4-phenylcoumarin (**5**); 5-*O*-β-D-glucopyranosyl-7-methoxy-3′,4′-dihydroxy-4-phenylcoumarin (**6**); 6′^′^-*O*-acetyl-5-*O-*β-D-galactopyranosyl-7,4′-dihydroxy-4-phenylcoumarin (**7**); 6′^′^-*O*-acetyl-5-*O-*β-D-galactopyranosyl-7,3′,4′-trihydroxy-4-phenylcoumarin (**8**); 6′^′^-*O*-acetyl-5-*O-*β-D-galactopyranosyl-7-methoxy-3′,4′-dihydroxy-4-phenylcoumarin (**9**); 5-*O*-[β-D-xylopyranosyl-(1 → 6)-β-D-glucopyranosyl]-7,4′-dimethoxy-4-phenylcoumarin (**10**).

## Author contributions

SC, RB conceptualization. SC, RB plant material acquisition. SC investigation. SC chemical analysis. SC, JN-S molecular analysis. SC data curation. SC, RB writing original draft. SC, RB, JN-S writing – review and editing.

### Conflict of interest statement

The authors declare that the research was conducted in the absence of any commercial or financial relationships that could be construed as a potential conflict of interest.

## Abbreviations

HL: *Hintonia latiflora*
HS: *Hintonia standleyana*
EC: *Exostema caribaeum*.
