# Supplementary figures and images for: Molecular Markers Associated With Chemical Analysis: A Powerful Tool for Quality Control Assessment of Copalchi Medicinal Plant Complex
Source: Front Pharmacol. 2018 Jun 22;9:666. doi: 10.3389/fphar.2018.00666 (PMC6024007; doi:10.3389/fphar.2018.00666)

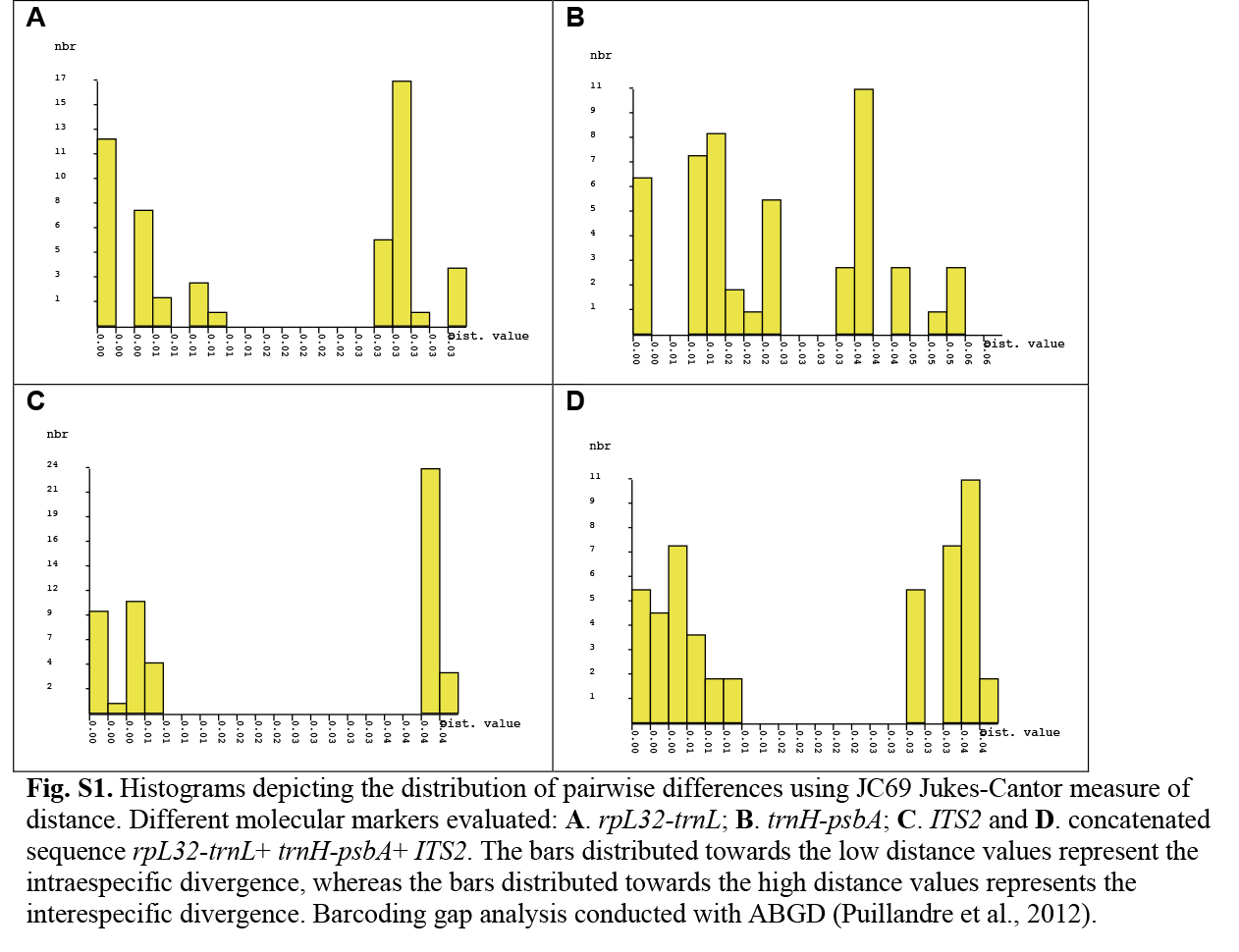

Supplement: Supplementary file 1 [file Image_1.tif]
